# Supplementary material for: Modeling glioblastoma heterogeneity as a dynamic network of cell states
Source: Mol Syst Biol. 2021 Sep 16;17(9):e10105. doi: 10.15252/msb.202010105 (PMC8444284; doi:10.15252/msb.202010105)
Supplement: Supplementary file 6 — Source Data for Figure 5 [file MSB-17-e10105-s004.zip › Figure5A_sourcedata/GSEA_3017/nosigdb_stateC.GseaPreranked.1622795002785/NB_KI_CLUSTER18.html]

Details for gene set NB\_KI\_CLUSTER18[GSEA]

|  || Dataset | state13017 |
| Phenotype | NoPhenotypeAvailable |
| Upregulated in class | na\_neg |
| GeneSet | NB\_KI\_CLUSTER18 |
| Enrichment Score (ES) | -0.55677927 |
| Normalized Enrichment Score (NES) | -2.6929429 |
| Nominal p-value | 0.0 |
| FDR q-value | 0.0 |
| FWER p-Value | 0.0 |
Table: GSEA Results Summary

  

Fig 1: Enrichment plot: NB\_KI\_CLUSTER18      
 Profile of the Running ES Score & Positions of GeneSet Members on the Rank Ordered List

  

| PROBE | GENE SYMBOL | GENE\_TITLE | RANK IN GENE LIST | RANK METRIC SCORE | RUNNING ES | CORE ENRICHMENT || 1 | IGFBP7 |  |  | 225 | -0.259 | -0.3749 | No |
| 2 | SOCS3 |  |  | 274 | -0.270 | -0.4380 | No |
| 3 | RRBP1 |  |  | 283 | -0.271 | -0.4305 | No |
| 4 | PHLDA1 |  |  | 295 | -0.274 | -0.4282 | No |
| 5 | NR2F2 |  |  | 313 | -0.283 | -0.4356 | No |
| 6 | COL4A2 |  |  | 353 | -0.300 | -0.4804 | No |
| 7 | LMNA |  |  | 376 | -0.308 | -0.4947 | No |
| 8 | FOS |  |  | 377 | -0.309 | -0.4703 | No |
| 9 | IGFBP5 |  |  | 384 | -0.311 | -0.4562 | No |
| 10 | TIMP1 |  |  | 399 | -0.320 | -0.4555 | No |
| 11 | CALD1 |  |  | 420 | -0.335 | -0.4641 | No |
| 12 | LGALS3 |  |  | 471 | -0.374 | -0.5224 | Yes |
| 13 | CAVIN1 |  |  | 481 | -0.387 | -0.5076 | Yes |
| 14 | PRSS23 |  |  | 510 | -0.438 | -0.5221 | Yes |
| 15 | CST3 |  |  | 520 | -0.452 | -0.5022 | Yes |
| 16 | CEBPD |  |  | 523 | -0.455 | -0.4697 | Yes |
| 17 | CYTOR |  |  | 530 | -0.468 | -0.4432 | Yes |
| 18 | IFITM3 |  |  | 533 | -0.477 | -0.4090 | Yes |
| 19 | COL6A1 |  |  | 534 | -0.479 | -0.3711 | Yes |
| 20 | COL4A1 |  |  | 545 | -0.507 | -0.3486 | Yes |
| 21 | TIMP3 |  |  | 553 | -0.530 | -0.3189 | Yes |
| 22 | SPARCL1 |  |  | 557 | -0.544 | -0.2812 | Yes |
| 23 | MT2A |  |  | 566 | -0.575 | -0.2498 | Yes |
| 24 | SPARC |  |  | 567 | -0.575 | -0.2043 | Yes |
| 25 | VIM |  |  | 578 | -0.674 | -0.1685 | Yes |
| 26 | NEAT1 |  |  | 579 | -0.683 | -0.1145 | Yes |
| 27 | CYR61 |  |  | 584 | -0.714 | -0.0650 | Yes |
| 28 | CCL2 |  |  | 595 | -1.066 | 0.0018 | Yes |
Table: GSEA details [plain text format]

  

Fig 2: NB\_KI\_CLUSTER18: Random ES distribution      
 Gene set null distribution of ES for **NB\_KI\_CLUSTER18**
